# The Effect of the Tumor Microenvironment on Lymphoid Neoplasms Derived from B Cells

**DOI:** 10.3390/diagnostics12030573

**Published:** 2022-02-23

**Authors:** Giuseppe Ingravallo, Roberto Tamma, Giuseppina Opinto, Tiziana Annese, Francesco Gaudio, Giorgina Specchia, Tommasina Perrone, Pellegrino Musto, Gerardo Cazzato, Emilio Bellitti, Saverio Capodiferro, Eugenio Maiorano, Domenico Ribatti

**Affiliations:** 1 Section of Pathology, Department of Emergency and Organ Transplantation (DETO), University of Bari Aldo Moro, Policlinico-Piazza G. Cesare, 11, 70124 Bari, Italy; emilio.bellitti@gmail.com (E.B.); eugenio.maiorano@uniba.it (E.M.); 2 Department of Basic Medical Sciences, Neurosciences and Sensory Organs, University of Bari Medical School, Policlinico-Piazza G. Cesare, 11, 70124 Bari, Italy; tiziana.annese@uniba.it (T.A.); domenico.ribatti@uniba.it (D.R.); 3 Haematology and Cell Therapy Unit, IRCCS-Istituto Tumori ‘Giovanni Paolo II’, Viale Orazio Flacco 65, 70124 Bari, Italy; g.opinto@oncologico.bari.it; 4 Hematology Section, Department of Emergency and Transplantation, University of Bari Medical School, 70124 Bari, Italy; francegaudio@gmail.com (F.G.); tommasinaperrone@virgilio.it (T.P.); pellegrino.musto@uniba.it (P.M.); 5 School of Medicine, University of Bari “Aldo Moro”, 70124 Bari, Italy; giorgina.specchia@uniba.it; 6 Department of Interdisciplinary Medicine, University of Bari Aldo Moro, Policlinico-Piazza G. Cesare, 11, 70124 Bari, Italy; capodiferro.saverio@gmail.com

**Keywords:** inflammatory cells, non-Hodgkin’s lymphomas, classical Hodgkin’s lymphomas, tumor microenvironment, tumor progression

## Abstract

Lymphomas are characteristic tumors surrounded by an inflammatory microenvironment. The cells of the microenvironment are essential for the growth and survival of neoplastic cells and are recruited through the effect of cytokines/chemokines. Lymphomas include heterogeneous groups of neoplasms infiltrating various lymphoid structures which may arise from B lymphocytes, T lymphocytes, and natural killer (NK) cells at various stages of their differentiation state. In this review article, we analyze the literature data concerning the involvement of the tumor microenvironment (TME) in the progression of lymphomas and the recent advances in the analysis of microenvironment components in the most common forms: some mature B cell lymphoma neoplasms and classic Hodgkin lymphomas. The complex crosstalk between the TME and tumor cells led to the discovery of many mechanisms usable as molecular-targeted therapy through the control of diverse elements of the TME, varying from inhibitors of angiogenic cytokines and their receptors to the regulation of cells’ activities and the novel immune checkpoint inhibitors.

## 1. Non-Hodgkin Lymphomas (NHLs) and Their Microenvironment

NHLs include heterogeneous groups of neoplasms infiltrating various lymphoid structures which may arise from B lymphocytes, T lymphocytes, and natural killer (NK) cells at various stages of their differentiation state and are characterized by a great tendency to disseminate towards extra-nodal locations [1]. About 25% of NHLs arise in extra-nodal locations and most of them are present in both nodal and extra-nodal sites. Based on their morphology, immunophenotype, genetic and clinical features, NHLs have been classified into more than 30 different types (Table 1) [2]. NHLs’ histologic features allow us to discriminate between a nodular and a diffuse pattern. In the first instance, the tumor cells aggregate to form large clusters. In the diffuse pattern, no evidence of nodularity or germinal center formation has been observed, and it is characterized by a profound impairment of lymph node architecture [3].

B cell-derived lymphomas may originate during any phase of normal B cell differentiation steps, even if most of them are derived from germinal center reactions [4]. 

The recent World Health Organization (WHO) classification exploits morphology, immunophenotype, and genetic findings to classify B cell lymphomas [5]. Clinical trials allowed us to distinguish the B cell lymphoma histological subtypes as indolent, aggressive, and very aggressive based on their typical clinical behavior [6,7]. The indolent lymphomas, whose overall survival is measured in years [8], represent about 40 percent of NHL and include the follicular lymphomas (FL), chronic lymphocytic leukemia/small lymphocytic lymphomas (CLL/SLL), a fraction of mantle cell lymphomas (MCL) cases, extra-medullary, nodal, and splenic marginal zone lymphomas (MZL), and lymphoplasmacytic lymphomas (LPL). The aggressive group include large B cell lymphomas, subdivided into anaplastic and primary mediastinal lymphomas, and various kinds of diffuse large B cell lymphomas (DLBCL). In this group, the survival of untreated patients is a few months but treatment may lead to definitive remissions and cure in a significant number of patients [9]. The highly aggressive group represents an NHL minority and is characterized by a survival of a few weeks if not adequately treated. The elected therapies for B cell NHL are chemotherapy, radiotherapy and immunotherapy, used either as monotherapies or as combined therapies [10]. 

Although their tumor initiation is related to the acquisition of oncogenic mutations in cells, the following tumorigenesis stages, including the growth, the progression and the metastatic process, are strongly influenced by the tumor microenvironment (TME) [11,12]. The TME is a complex environment surrounding the cancer cells that includes cellular and extracellular components and a vascular network [13], which are also involved in the determination of therapeutic efficacy [14]. Hanahan and Weinberg modified the hallmarks of cancer expanding them from six to ten, underlining the importance of the TME in cancer development [15]. Lately, experimental data have demonstrated that the TME, the factors delivered into it, the stroma, and the agents inducing DNA-damage, influence positively the neoplastic risk [16]. The equilibrium of cytokines in a tumor is crucial for regulating the type and expansion of its inflammatory infiltrate [17,18].

## 2. DLBCL

DLBCL is the largest group of NHLs, representing 49% of B cell cancers worldwide [19]. The median age of prevalence is seventy years, although it has been diagnosed in young people and rarely in children, with a slight male prevalence [20]. DLBCL is characterized by a high heterogeneity at both the clinical and biological levels because it arises from germinal center B cells at different stages of differentiation associated with recurrent genetic modifications which contribute to the molecular pathogenesis of the disease [21]. On these bases, the DLBCL classification results are very complex and constantly evolving due to heterogenic variants in consideration to morphology, phenotype, genetic anomalies, prognosis and clinical features [22]. DLBCL tumor mass may grow in lymph nodes and/or in other multiple external sites, but the gastrointestinal tract constitutes the most frequent primary tumor site [23]. CD68^+^ macrophages, tryptase^+^ mast cells and microvascular density (MVD) have been evaluated in samples derived from DLBCL patients subdivided into two groups. The first group included patients who achieved a complete remission (responders), and the second included patients who never achieved a complete remission or incomplete remission after first-line chemotherapy, and who relapsed within six months (non-responders) [24]. A higher number of both CD68^+^ cells and microvessels in the non-responders group compared to the responders group has been observed [24]. In DLBCL, a high expression of CD68^+^ cells has been correlated with a poor prognosis [25]. The higher percentage of tryptase^+^ mast cells found in the non-responders’ group when compared with the responders’ group positively correlated with the MVD [24], indicating the important role of mast cells in promoting and sustaining tumor angiogenesis in DLBCL. Bulky and residual tumors are considered to increase the risk of relapse in DLBCL patients [26]. To investigate the complex relationships occurring between immune cells, stromal cells, endothelial cells and the tumor cells, the involvement of T cells in the control of bulky and non-bulky DLBCL development and their correlation with mast cells and MVD has been estimated [27]. A significant reduction in CD3^+^ cells in the TME of bulky compared to non-bulky disease has been reported, suggesting the loss of the immune control resulting in an increased cell proliferation, and consequently to a large tumor cell-mass in bulky DLBCL [27]. Moreover, the positive correlation between the percentages of CD3^+^ cells and tryptase^+^ mast cells reveals a complex link between T cells and mast cells in the induction of tumor angiogenesis in DLBCL [27]. DLBCL, based on its gene expression profile could be classified as germinal center (GC) B-like DLBCL (GCB) and an activated B-like DLBCL (ABC), with unique gene expression signatures such as constitutive activation of nuclear factor kB (NFkB) in ABC DLBCL, and somatic mutations of polycomb repressor 2 complex gene EZH2 in GCB DLBCL [28]. Furthermore, the GCB group expresses high levels of BCL6 and responds better to conventional chemotherapy, whereas the ABC group has lower levels of BCL6 and tends to be refractory to chemotherapeutic treatment [29]. The number of tumor and T cells present in the TME determining the percentages of CD20^+^ and CD3^+^, respectively, in the DLBC GCB and -non-GCB subgroups has been estimated [30]. A lower CD20^+^ count was observed in patients with a non-GCB immunophenotype, high LDH, splenomegaly and an IPI ≥ 3, while CD3^+^ cells were lower in patients with bulky disease, an IPI ≥ 3 and disease stage of 3–4 [30]. 

Constitutively activated STAT3 is correlated with a more advanced clinical stage and overall poor survival rate of people with DLBCL [31,32]. In addition, STAT3 is strongly activated in ABC-DLBCL and BCL6-negative normal germinal center B cells representing both the second oncogenic pathway in ABC-DLBCL and an additional therapeutic target for treatment [33]. The DLBCL GCB and ABC subgroups of patients have been compared and by means of RNAscope technology a significantly higher number of STAT-3-expressing cells in ABC group as compared to GCB has been shown [34]. Tumor vessels in ABC samples appeared lined by endothelial cells expressing both FVIII and STAT3 signals, while in GCB samples, only few vessels co-expressed FVIII and STAT3 [34]. A higher Ki67 expression in tumor cells and a higher number of CD163^+^ macrophages in ABC patients as compared with GBC ones has been observed, together with a high density of CD3^+^ and CD8^+^ cells, which correlated with STAT3 expression and microvascular density [35]. In the DLBCL TME, the prognostic value of the CD4/CD8 ratio has been associated with both better and worse survival in different studies [36]. No variation in CD4^+^ cells in ABC with respect to GCB has been observed but a higher CD8^+^ cell infiltrate in the ABC group associated with a decreased CD4/CD8 ratio [35]. In addition, a higher STAT3 expression is associated not only with CD8^+^ cells but also with a higher M2 TAM cell infiltration [35].

## 3. MCL

MCL represents around 2–10% of NHLs in adults with a median age at diagnosis of 65 years old, principally observed in men more than in women, at a 3:1 ratio. It originates in the lymph nodes although metastasis in the bone marrow, spleen, and gastrointestinal tract are frequently present [37]. The WHO 2016 update about lymphomas distinguished two MCL main subgroups according to the clinical presentation and molecular mutations: classical- and non-nodal-MCL [38]. The first one is characterized by an unmuted immunoglobulin variable region heavy chain (IgHV) and SOX11^+^ with a blastoid or pleomorphic morphology associated with an aggressive outcome. The second one is characterized by hypermutated IgHV and SOX11-, with an indolent disease course. In our study, MCL samples were divided into three histological groups based on SOX11-IHC positivity: “negative” with no staining and 0% of SOX11^+^ cells; “light” with a weak-moderate staining and 1–39% of SOX11^+^ cells; and “strong” with a moderate-strong staining and ≥40% of SOX11^+^ cells [39]. Higher CD4^+^ and CD8^+^ T-lymphocyte infiltrates in MCL lymph node biopsies belonging to the strong group compared to the other groups have been demonstrated and correlated with SOX11 intensity and increased angiogenesis [39]. In the strong group, characterized by worse outcomes, CD8^+^ cells could be involved in the activation of tumor immune evasion processes because CD8^+^ cells promote the secretion of prostaglandin E2 which consequently induces tumor escape via the Fas signaling pathway [40,41]. Reduced CD68^+^ and CD163^+^ TAM in MCL in the strong group compared to the other groups inversely correlated with increased SOX11^+^ cells and angiogenesis [39]. The p53 expression results were negative or very low in all the samples. The functional loss of p53 induces excessive inflammatory reactions that in turn sustain tumor growth and progression [42]. Although the literature data indicate that STAT3 is constitutively activated and acts by decreasing SOX11, the evaluation of STAT3 mRNA expression did not reveal any variation among the three analyzed MCL group of patients. 

## 4. MZL

MZL is the second most common subtype of indolent B cell NHL, accounting for 10% of total NHL [43,44,45]. It evolves starting from memory B cells residing in a micro-anatomic compartment of the secondary lymphoid follicles. This compartment is named as the “marginal zone” and it is in mucosa-associated lymphoid tissues (MALT) and in the spleen [46]. The WHO classified MZL as extra-nodal MZL of MALT type, splenic MZL (SMZL), and nodal MZL (NMZL) [47]. About one-third of MZL cases localize it the stomach, but other localization sites include gastrointestinal sites as well as in salivary glands, ocular adnexa, thyroid, lung, skin, breast, and liver [48,49]. MALT lymphoma grows in organs lacking in lymphoid tissue and in which B cells accumulate as a consequence of chronic inflammatory stimuli including *Helicobacter pylori*, *Chlamydophila psittaci*, *Borrelia burgdorferi* infections [50,51,52], as well as in chronic C hepatitis or autoimmune diseases, including Sjogren Syndrome and Systemic Lupus Erythematous [53]. MALT lymphomas in the TME in addition to B cells present the infiltration of T cells, macrophages, and neutrophils. In MZL-MALT lymphoma specimens the inflammatory cells infiltrating the TME have been analyzed and quantified [13]. The results indicate an increased number of CD3^+^, CD4^+^ and CD8^+^ lymphocytes, CD68^+^, CD163^+^ macrophages and tryptase^+^ mast cells in the MALT group samples compared to the healthy ones. In addition, a higher number of CD34^+^ vessels have been demonstrated in MALT lymphoma samples in a positive correlation with CD8^+^ cells, underlying the important role of these cells in tumor angiogenesis. Furthermore, the number of CD8^+^ cells correlated with M2-type macrophages, while tryptase^+^ mast cells correlated with CD4^+^ cells, indicating the complex crosstalk between TME-infiltrating cells [13].

## 5. Classical Hodgkin’s Lymphoma and Its Microenvironment

Classical Hodgkin’s lymphoma (cHL) is one of the most common lymphomas positive for the presence of large multi- and mono-nucleated cells named Reed–Sternberg (RS) and Hodgkin (H) cells, respectively [5]. RS and H cells correspond to just 1% to 10% of total tumor mass; the remaining 90% is composed of tumor inflammatory cells, including T and B lymphocytes, plasma cells, histiocytes/macrophages, granulocytes, eosinophils, mast cells, and mesenchymal stromal cells (MSCs) [54].

The study of microdissections has revealed that RS and H cells carry Ig genes somatic hypermutations and clonal Ig rearrangements, suggesting their origin from pre-apoptotic germinal center (GC) B cells [55]. However, RS cells present an unusual immunophenotype characterized by the absence of B cell markers, associated with possible co-expression of molecules of various hematopoietic lineages [56]. 

Different cytokines/chemokines, including IL-5, IL-7, IL-8, CCL5 (RANTES), CCL17 (TARC), CCL20 and CCL28, produced by RS cells, H cells and recruited immune cells, influence the TME composition [57]. T cells secrete IL-3, IL-10 and RANKL: IL-3 influences the formation of the inflammatory infiltrate and supports the neoplastic cell growth [58], whereas IL-10 promotes strong anti-inflammatory properties [59] and RANKL contributes to the activation and survival of dendritic cells [60]. Stromal cells may secrete CCL11, which attracts eosinophils and Th2 cells, IL-7 and CCL5 are involved in the growth or survival of RS cells, while dendritic cells secrete TARCs, which are involved in the recruitment of Th2 cells and regulatory T cells [57]. Macrophages secrete cytokines involved in tumor progression such as macrophage migration inhibitory factor (MIF), IL-8 and TNFα. In particular, MIF may contribute to the proliferation of RS cells in the TME [61] and IL-8 may increase neutrophilic recruitment [62]. 

Moreover, mast cells may increase the survival signals of tumor cells through CD30L [63]. The T component was composited by CD4^+^ Th cells, CD4^+^ T-regulatory (Tregs) and CD8 cytotoxic T lymphocytes (CTL). CD4^+^ T cells are sometimes in close contact with RS cells as they can form rosettes around the neoplastic blasts [64,65]. In the immune synapse between RS cells and CD4^+^ T cells, the interactions between TCR-MHCII and CD2-CD58 are needed for T cell activation, while the CD2-C58 axis is associated with cell adhesion and rosette formation [66]. 

The prevalent Th phenotype present in the cHL TME is the Th2 [62]. However, a recent study found the predominance of an activated, proliferative and pro-inflammatory cytokine-secretory phenotype, typical of Th1 cells [67]. The CD8^+^ T cells are a proportion of T-cell infiltrate of HL and are not in close contact with the tumor cells [68]. However paradoxically, an increased numbers of cytotoxic T^+^ cells for cytotoxic granule-associated RNA-binding protein (TIA1) in the TME correlated with poor outcomes [69,70].

In the TME, there is also an accumulation of Treg, expressing [71] factor forkhead box P3 (FOXP3) [72]. In cHL patients, a higher number of intra-tumoral FOXP3^+^ Treg cells was associated with longer DFS and OS, even in multivariate analyses [71]. 

Tumor-Associated Macrophages (TAM) are present in cHL. According to a topographical model, in the TME abundant PD-L1+TAMs and PD-1^+^ CD4 T cells were observed, which were in contact with PD-L1^+^ tumor cells. These observations supported a possible role also of the macrophages in the mechanism of action of checkpoint inhibitor therapy [73]. 

A macrophage gene signature correlated with the failure of primary treatment and in an independent cohort of patients it has been demonstrated by immunohistochemistry that a higher number of CD68^+^ TAM was associated with shortened survival and with the outcome of secondary treatments, such as autologous stem-cell transplantation [74]. Other studies, but not all [75,76,77], have confirmed the relationship among TAMs and lower outcomes after upfront treatment [78,79,80,81]. Moreover, the molecular characterization of HR cells reported as neoplastic clone the over-expression of colony-stimulating factor 1 receptor (CSF1R), a gene of the macrophage signature, and the latter gene was associated with primary treatment failure [82].

A correlation between the the number of M1 TAMs and favorable prognosis in the mixed cellularity subtype of cHL has been reported [83].

Large quantities of non-malignant B cells are present in the microenvironment of HL, but their role is still not well established [84].

The association between the number of mast cells and survival of patients with cHL is controversial. A significant association between high mast cell counts and poorer EFS, as well as OS in mixed cellularity but not in nodular sclerosis histological subtype, has been demonstrated [85].

## 6. Concluding Remarks

The TME includes a rich cellular component in which secreted cytokines and chemokines are involved in the regulation of tumor initiation as well as its progression and metastasis; furthermore, the constitution of the TME also has important effects on therapeutic efficacy. The complex crosstalk between the TME and tumor cells led to the discovery of many mechanisms usable as molecular-targeted therapies through the control of diverse elements of the TME, varying from inhibitors of angiogenic cytokines and their receptors to the regulation of cells activities and the novel immune checkpoint inhibitors. The mechanisms of tumor evasion and resistance frequently reduce the efficacy of these new therapeutic approaches and consequently they currently constitute a major focus of research. 

## Figures and Tables

**Table 1:** Classification of NHLs.

Indolent lymphomas	Follicular lymphoma B-chronic lymphocytic leukemia/small lymphocytic lymphoma Lymphoplasmacytic lymphoma Marginal zone lymphoma (nodal, extra-nodal, splenic) T/natural killer large cell granular lymphocyte leukemia T-chronic lymphocytic leukemia/prolymphocytic leukemia
Aggressive lymphomas	Mantle cell lymphoma Diffuse large B cell lymphoma Peripheral T-cell lymphoma (unspecified) Peripheral T-cell lymphoma (angioimmunoblastic, angiocentric T/natural killer cell, hepatosplenic γ/δ, intestinal T cell lymphoma) Anaplastic large cell lymphomas
Highly aggressive lymphomas	Precursor T or B lymphoblastic leukemia/lymphoma. Burkitt and Burkitt-like lymphoma. Adult T-cell leukemia/lymphoma (HTLV-1+)

## Data Availability

Not applicable.
